# Effects of Apigenin and Astragalus Polysaccharide on the Cryopreservation of Bull Semen

**DOI:** 10.3390/ani11061506

**Published:** 2021-05-22

**Authors:** Hongtao Wang, Ping Lu, Chongshan Yuan, Jing Zhao, Hongyu Liu, Wenfa Lu, Jun Wang

**Affiliations:** 1 Joint Laboratory of Modern Agricultural Technology International Cooperation, Ministry of Education, Jilin Agricultural University, Changchun 130118, China; hongtao2021@yeah.net (H.W.); luping100419@163.com (P.L.); 18844146800@163.com (C.Y.); jlndfztd@163.com (J.Z.); jlndlhy0133@163.com (H.L.); 2 Key Lab of Animal Production, Product Quality and Security, Ministry of Education, Jilin Agricultural University, Changchun 130118, China; 3 College of Animal Science and Technology, Jilin Agricultural University, Changchun 130118, China

**Keywords:** apigenin, astragalus polysaccharide, bull semen, cryopreservation

## Abstract

**Simple Summary:**

Oxidative stress in mammalian sperm can be induced during cryopreservation; therefore, it is important to inhibit oxidative stress to maintain sperm motility during cryopreservation. The present study was performed to investigate the effects of supplementing apigenin (AP), astragalus polysaccharide (APS), or their combination on an extender for bull semen freezing. Sperm viability, motility, and motion parameters; acrosome integrity and membrane integrity; endogenous antioxidant indices; and reactive oxygen species (ROS) and malondialdehyde (MDA) levels were evaluated after semen thawing. Our results indicated that 0.2 mmol/L AP or 0.5 mg/mL APS improved the cryopreservation of bull sperm; moreover, their combination in the extender could further improve the protective effects on bull sperm post-thaw.

**Abstract:**

The purpose of this study was to determine the effects of apigenin and astragalus polysaccharides on the cryopreservation of bovine semen. Apigenin, astragalus polysaccharides, or their combination were added to a frozen diluent of bovine semen. Afterwards, Computer Assisted Semen Analysis **(**CASA), membrane functionality, acrosome integrity, mitochondrial integrity, CAT, SOD, GSH-Px, MDA, and ROS detection were conducted. The results showed that adding 0.2 mmol/L AP or 0.5 mg/mL APS could improve the quality of frozen sperm. Compared to 0.2 mmol/L AP alone, the combination of 0.2 mmol/L AP and 0.3 mg/mL APS significantly increased the total motility (TM), average path distance (DAP), straight line distance (DSL), average path velocity (VAP), curvilinear velocity (VCL), wobble (WOB), and sperm CAT and SOD levels (*p* < 0.05), while reducing the ROS and MDA levels (*p* < 0.05). These results indicated that the addition of 0.2 mmol/L AP or 0.5 mg/mL APS alone has a protective effect on the freezing of bovine semen. Compared to the addition of 0.2 mmol/L AP, a combination of 0.2 mmol/L AP and 0.3 mg/mL APS could further improve the quality of frozen semen.

## 1. Introduction

Artificial insemination (AI) accounts for an increasing proportion of cattle reproduction [1]. The development of AI involves increasingly stringent requirements on the quality of frozen semen [2]. Although the quality of semen after thawing has improved in the past few decades, about 50% of sperm are fixed or destroyed due to freezing and thawing, resulting in a reduced fertilization ability [3]. The oxidative stress caused by the production of reactive oxygen species (ROS) during the freezing and thawing process was found to be the main reason for the decreased semen motility after thawing. As mammalian sperms lack part of the cytoplasm, their antioxidant capacity is not sufficient to resist the lipid peroxidation (LPO) caused by the ROS-induced polyunsaturated fatty acids that are bound to sperm phospholipids [4]. Due to the antioxidant enzymes in semen, such as glutathione peroxidase (GSH-Px), catalase (CAT), and superoxide dismutase (SOD), concentrations are very important. As a mammalian sperm cytoplasm has a relatively high concentration of polyunsaturated fatty acids (PUFA), it lacks sufficient natural antioxidant reserves. Therefore, the addition of natural antioxidants to the frozen semen dilution has become necessary to improve sperm motility after thawing. Antioxidants can balance ROS and reduce LPO levels to improve the freezing resistance of semen and improve the quality of melted semen. Antioxidants have been widely used in frozen semen [4,5,6]. Plant-derived compounds are considered to be important sources of antioxidants because they produce almost no side effects and are easily available in nature [7].

Flavonoids are widely present in nature. These compounds act as free radical scavengers and antioxidants, and have antimutagenic, anti-inflammatory, and antiviral effects [8,9,10,11]. Apigenin (AP) (APEBIO Co., Ltd., Houston, Texas, USA), a flavonoid, is widely distributed in a plant [10]. Compared to that of other structurally related flavonoids, it has a lower inherent toxicity to normal cells [12,13]. AP penetrates cells and binds to DNA to form an apigenin-DNA complex, protecting the DNA from oxidative stress in the cell [14]. Recent studies have demonstrated that adding AP to a frozen diluent of swine semen could increase sperm motility and the antioxidant enzyme content and reduce the concentration of the LPO product malondialdehyde after thawing [2]. Astragalus polysaccharide (APS) (Shanghai Yuanye Biological Co., Ltd., Shanghai, China) can be considered as an antioxidant as there is research that shows that APS can inhibit mitochondrial damage by scavenging ROS [15]. Recent studies have found that APS could reduce the oxidative damage of cardiomyocytes caused by free radicals and lipid peroxides as well as reduce membrane damage. Furthermore, APSs have recently been found to have antibacterial and antiviral properties and can be used as immune enhancers [16]. Due to the various properties of APS, it can be used for the cryopreservation of pig semen, and studies have shown that it can improve semen vitality and antioxidant levels [17].

Different antioxidants have a synergistic effect on the removal of active oxygen [18,19]. AP and APS, as two different antioxidants, have been shown to improve semen quality through different action pathways and action times [17,20]. However, neither of the two substances have been used before in the freezing of bovine semen. The purpose of this study was to explore the effects of AP, APS, and their combination on the freezing of bovine semen.

## 2. Materials and Methods

### 2.1. Experimental Design

In this study, pooled semen was extended using a Tris extender with different levels of AP (0, 0.2, 0.4, 0.6, and 0.8 mmol/L) and APS (0, 0.1, 0.3, 0.5, and 0.7 mg/mL) or a combination of AP and APS (0.2 mmol/L AP, 0.2 mmol/L + 0.1 mg/mL, 0.2 mmol/L + 0.3 mg/mL, 0.2 mmol/L + 0.5 mg/mL, and 0.2 mmol/L + 0.7 mg/mL). Each experiment was repeated at least three times.

### 2.2. Animals

This study was conducted at Jilin Agricultural University in P.R. China. Experimental Animal Welfare and Ethics Committee of Jilin Agricultural University approval was gained, the number is 20200803001. Four bulls (4 years old) were used for semen collection. The uniformity of the feed, housing, and light conditions were ensured. The bull feed was composed of 45% corn, 32% soybean cake, 5% wheat bran, 5% rice bran meal, 6% soybean germ meal, 2% molasses, and 5% bull special premix. In terms of nutritional indicators, the bull feed had approximately 20% crude protein, 13% moisture, 7% crude ash, 0.6–0.7% calcium, 0.6–0.7% total phosphorus, and 0.8–1% sodium chloride. The bulls had free access to water and salt, and no additional antioxidants were present in the feed.

### 2.3. Bull Semen Collection

Semen samples were collected using an artificial vagina twice a week per bull, for 12 weeks. The criteria for cryopreservation were as follows: the semen samples were sent to the laboratory within 30 m. The semen was held in a water bath at 37 °C while the sperm concentration was estimated using a calibrated spectrophotometer and the motility of sperm was subjectively evaluated using microscopy, at a concentration of at least 1 × 10^9^ spermatozoa/mL, a sperm motility ≥70%, and an abnormality ≤15%. Healthy ejaculates were used in the experiments. After the initial assessment, the semen samples were mixed to eliminate individual differences.

### 2.4. Basic Extender

The basic extender was slightly modified from that described by Tarig et al. [21], as shown in Table 1. All of the chemicals were placed into a capacity bottle and double evaporative water was used to stabilize the capacity to 100 mL, a magnetic stirrer was used to stir for 2 h, then 20% egg yolk and 3% glycerin were added and stirred again for two hours to completely dissolve. The basic diluent was divided evenly and AP or APS of different concentrations were added.

### 2.5. Semen Processing

After the quality evaluation, the samples were diluted and incubated in a water bath at 37 °C for 30 min for the full absorption of AP and APS by the sperm membrane. After that, the samples were packed in 0.25 mL straws (IMV Co., Ltd, L’Aigle, Paris, France) with 8 × 10^6^ sperm/straws, then cooled from 37 °C to 4 °C for 2 h, as previously described, and subsequently cooled from 4 °C to −140 °C for approximately 8 min, using a turbo freezer (Minitube Co., Ltd, Munich, Bavaria, Germany). After that, the straws were transferred to a liquid nitrogen tank (−196 °C) and stored for 1 month before detection.

### 2.6. Evaluation of Post-Thawed Sperm

#### 2.6.1. Computer Assisted Semen Analysis

The kinematic parameters of sperm were analyzed using a sperm analyzer (developed jointly by Hamilton and IMV, IVOS II, 10871, 6 October 2017). Two straws were thawed by immersion in a water bath at 37 °C for 30 s. Each sample was analyzed at least three times. Five microliters were detected for each straw, and four fields were randomly examined. The total motility (TM), average path velocity (VPA), straight line velocity (VSL), curvilinear velocity (VCL), amplitude of lateral head displacement (ALH), beat/cross frequency (BCF), straightness (STR), linearity (LIN), wobble (WOB), distance of average path (DAP), straight line distance (DSL), and curvilinear distance (DCL) were recorded.

#### 2.6.2. Acrosome Integrity

**Figure 1:**
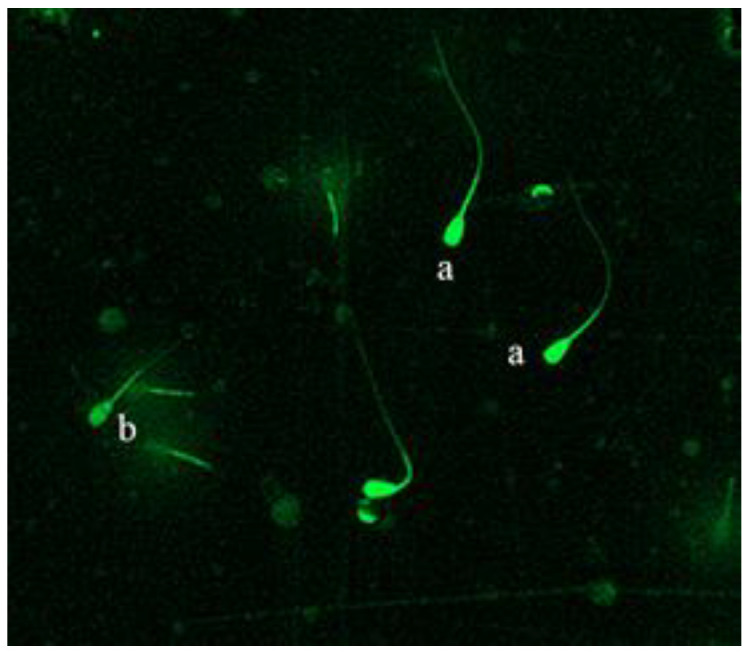
After fluorescent staining with FITC-PSA, live sperms were marked by green fluorescence (**a**) and dead sperms were marked by light green (**b**).

The sperm acrosome integrity was slightly modified from that described by Masoudi et al. [22]. The sample was centrifuged, and the resultant sperm pellet was obtained. It was then equilibrated in 96% ethanol for 10 min. Afterwards, the sperm was placed on a glass slide and fluorescein isothiocyanate-conjugated pea lectin (PSA-FITC) (sigma) was added. The slides were incubated for 20 m, and glycerol was added to fix the sperm onto the slide. At least 5 fields of view were observed through a fluorescence microscope (400× magnification) (Cytation 5 imaging reader, Bio Tek Co., Ltd, Winusky, Vermont, USA). The percentage of sperm with abnormal acrosomes was recorded by counting a total of 200 sperm under visualized microscopically. (see Figure 1).

#### 2.6.3. Plasma Membrane Integrity

**Figure 2:**
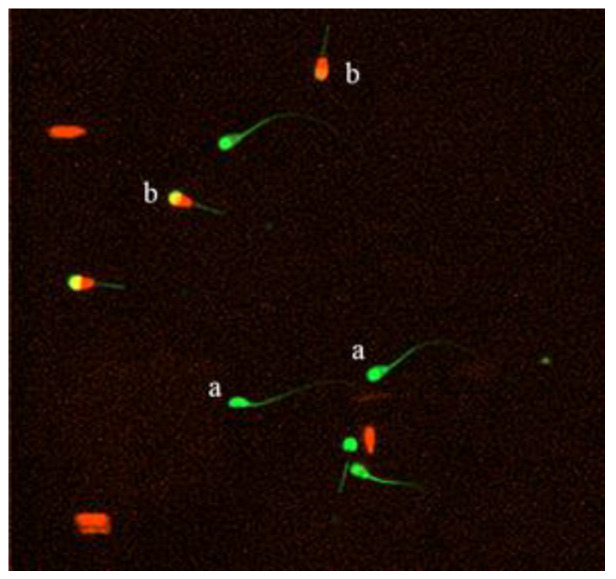
After double fluorescent staining with CFDA/PI, live sperms were marked by green heads (**a**) and dead sperms were marked by red heads (**b**).

The method of measuring sperm plasma membrane integrity used was slightly modified according to the method in an article published by R.A. Harrison et al. [23]. Using the double staining method of carboxy fluorescein diacetate (CFDA) (Andy Forno BiotechnologyCo., Ltd. Wuhan, China) and propidium iodide (PI) (Coolaber), 0.46 mg of CFDA was dissolved in 1 mL of dimethyl sulfoxide and 0.5 mg of PI was dissolved in 1 mL of normal saline. It was stored at −20 °C and protected from light. Afterwards, 20 μL of CFDA and 10 μL of PI were dissolved in 1 mL of PBS with a pH of 7.4 and 0.01 mol/L. A 20-microliter semen sample was then taken and an 80-microliter staining solution was added. The cells were incubated at 37 °C for 10 min and washed with PBS. After washing, a 10-microliter sample and was taken and at least 5 fields of view were observed through a fluorescence microscope (400× magnification) (Cytation 5 imaging reader, Bio Tek Co., Ltd, Winusky, Vermont, USA). The percentage of sperm with abnormal acrosomes was recorded by counting a total of 200 sperm under visualized microscopically. (see Figure 2).

#### 2.6.4. Mitochondrial Activity

**Figure 3:**
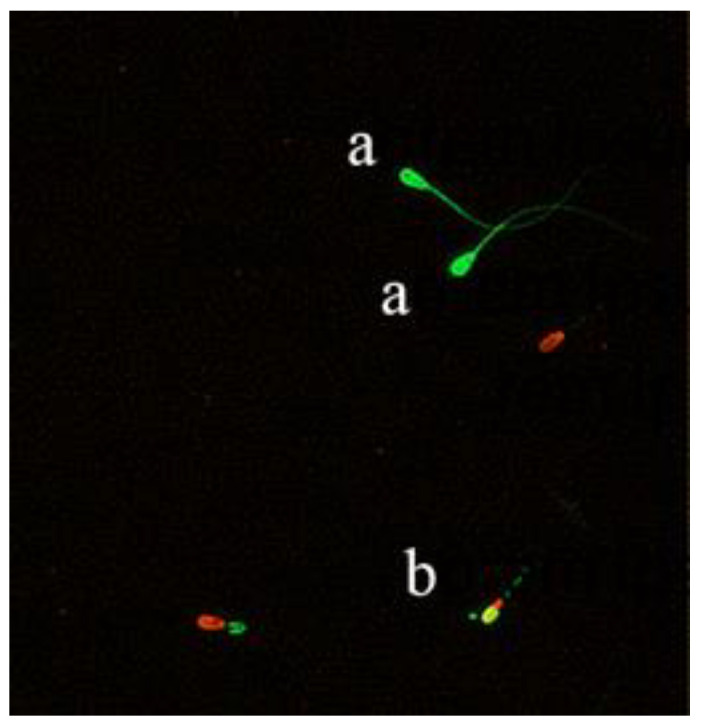
After double fluorescent staining with Rh123/PI, live sperms were marked by green heads (**a**) and dead sperms were marked by red heads (**b**).

The method of measuring sperm mitochondrial activity was slightly modified according to the method in an article published by Shahverdi et al. [24]. The mitochondrial activity of sperm was evaluated using double fluorescent staining with rhodamine 123 (Rh123) (Coolaber) and PI. Rh123 was dissolved with Me2SO and stored in the dark, while PI was dissolved in a physiological saline. The semen was then mixed with Rh123 and PI, incubated for 20 min, taken out and centrifuged to remove the supernatant, and then was added to PBS to mix the semen. Afterwards, the sample was centrifuged again to wash away the impurities and at least 5 fields of view were observed through a fluorescence microscope (400× magnification) (Cytation 5 imaging reader, Bio Tek Co., Ltd, Winusky, Vermont, USA). The percentage of sperm with abnormal acrosomes was recorded by counting a total of 200 sperm under visualized microscopically. (see Figure 3).

#### 2.6.5. Endogenous Antioxidant Indices Detection in the Frozen–Thawed Semen

The determination of various endogenous antioxidant enzymes (superoxide dismutase, glutathione peroxidase, and catalase) was carried out using an enzyme-linked immunoassay kit (Shanghai Enzyme Biotechnology Co., Ltd., China), according to the manufacturer’s instructions. The sample to be tested was mixed with a sample diluent. After incubation, the enzyme-labeling reagent and stop solution were added in sequence. The resulting mixture was then placed into a microplate reader for analysis.

#### 2.6.6. MDA and ROS Concentration Determination in Post-Thawed Semen

A bull MDA and ROS ELISA assay kit was used to determine the concentrations of MDA and ROS (Shanghai Enzyme Biotechnology Co. Ltd., China). The bovine semen was centrifuged repeatedly to destroy the sperm’s structure. Next, 40 μL of diluent was added to the sample, and placed onto a shaker to mix the semen and diluent thoroughly. It was then incubated in a 37 °C incubator for 30 min, washed with a washing solution 5 times, 50 μL of enzyme-labeled reagent was added, incubated again in a 37 °C incubator for 30 min, and washed 5 times after equilibration. Two kinds of diluent shake were then added and mixed well. The entire procedure was performed for 10 min at 37 ℃ in the dark. After adding the stop solution, the absorbance was measured using a microplate reader at a wavelength of 450 nm, and the content of MDA and ROS was calculated according to the standard curve.

### 2.7. Statistical Analyses

All results were expressed as mean ± SEM. The mean values of the sperm characteristics, movement characteristics, GSH-Px, CAT, SOD, ROS, and MDA concentrations, acrosome integrity, mitochondrial activity, and membrane integrity were compared using Statistical Product and Service Solutions (SPSS 22.0; SPSS, Chicago, IL, USA) Duncan’s multiple range test by ANOVA procedure; *p* < 0.05 was considered statistically significant.

## 3. Result

### 3.1. Effect of Different Concentrations of AP and APS on Several Kinematic Parameters of Bovine Semen Samples after Thawing

#### 3.1.1. Effect of Different Concentrations of AP on Several Kinematic Parameters of Frozen Bovine Semen

The results are shown in Table 2. Adding different concentrations of AP could improve the kinematic parameters of frozen semen. A concentration of 0.2 mmol/L significantly improved the kinematic ability of the TM, DAP, DSL, VAP, VSL, LIN, and WOB (*p* < 0.05). Simultaneously, 0.2 mmol/L AP relieved the DCL and VCL levels (*p* < 0.05). Although adding 0.4 mmol/L AP also had a certain effect, it was not as good as the state of the sperm when 0.2 mmol/L was added.

#### 3.1.2. Effect of Different Concentrations of APS on Several Kinematic Parameters of Frozen Bovine Semen

The results are shown in Table 3. Adding different concentrations of APS improved the kinematic parameters of semen freezing. When the concentration was 0.5 mg/mL, the sperm’s TM, DAP, DSL, VAP, VSL, LIN, WOB levels were significantly increased, and the DCL and VCL levels were simultaneously relieved (*p* < 0.05). Although the addition of 0.5 and 0.7 mg/mL APS was also significantly different from the control group in some kinematic parameters (*p* < 0.05), generally, the best effect was achieved when 0.5 mg/mL APS was added.

### 3.2. Effects of AP and APS at Different Concentrations on Sperm Plasma Membrane Integrity, Acrosome Integrity, and Mitochondria Activity after Thawing

#### 3.2.1. Effects of AP at Different Concentrations on Sperm Plasma Membrane Integrity, Acrosome Integrity, and Mitochondria Activity

**Figure 4:**
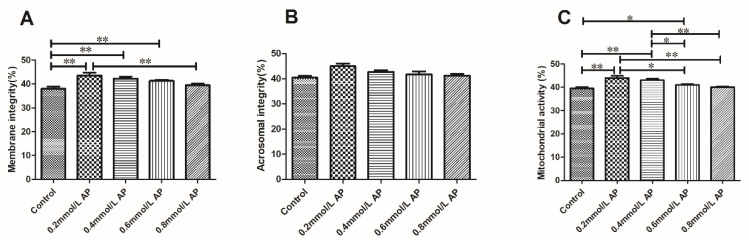
Effect of different concentrations of AP on the integrity of the plasma membrane (**A**), acrosome (**B**), and mitochondria (**C**) after the thawing of bovine semen. The different asterisks represent significant differences among the groups (* *p* < 0.05; ** *p* < 0.01).

The results of plasma membrane, acrosome integrity, and mitochondria activity assays following a supplementation with 0, 0.2, 0.4, 0.6, and 0.8 mmol/L AP are summarized in Figure 4. The mitochondria activity was significantly higher in the 0.2 mmol/L group (*p* < 0.05), and both the plasma membrane and the acrosome integrity were also improved in the AP 0.2 mmol/L (*p* < 0.05) group, when compared to the others.

#### 3.2.2. Effects of APS at Different Concentrations on Sperm Plasma Membrane Integrity, Acrosome Integrity, and Mitochondria Activity

**Figure 5:**
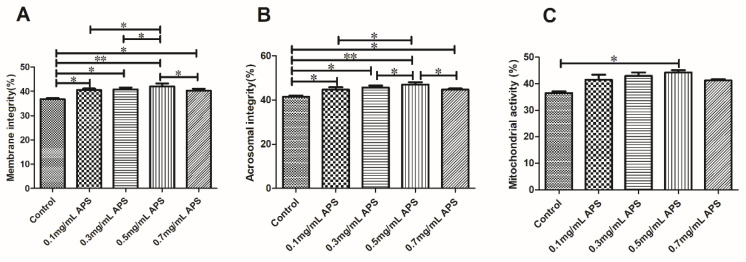
Effect of different concentrations of APS on the integrity of the plasma membrane (**A**), acrosome (**B**), and mitochondria (**C**) after the thawing of bovine semen. The different asterisks represent significant differences among the groups (* *p* < 0.05; ** *p* < 0.01).

The results of plasma membrane, acrosome integrity, and mitochondria activity assays following a supplementation with 0, 0.1, 0.3, 0.5, and 0.7 mg/mL APS are summarized in Figure 5. The mitochondria activity, plasma membrane, and acrosome integrity were significantly higher in the 0.5 mg/mL group (*p* < 0.05).

### 3.3. Antioxidant Enzymes, ROS, and MDA Content in Semen after Thawing

#### 3.3.1. Effect of AP on the Antioxidant Enzyme Level in Sperm

**Figure 6:**
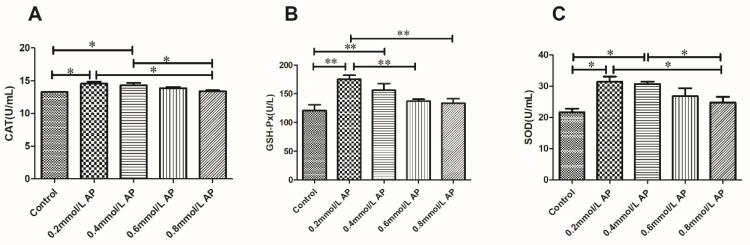
Effect of different concentrations of AP on the antioxidant enzyme activity in bull semen after thawing (**A**–**C**). The different asterisks represent significant differences among the groups (* *p* < 0.05; ** *p* < 0.01).

The results of the CAT, GSH-Px, and SOD assays after semen cryopreservation in 0, 0.2, 0.4, 0.6, and 0.8 mmol/L AP are shown in Figure 6. The CAT, GSH-Px, and SOD levels in the AP 0.2 mmol/L groups retained better activity (*p* < 0.05) than the other groups.

#### 3.3.2. Effects of APS on the Antioxidant Enzyme Level in Sperm

**Figure 7:**
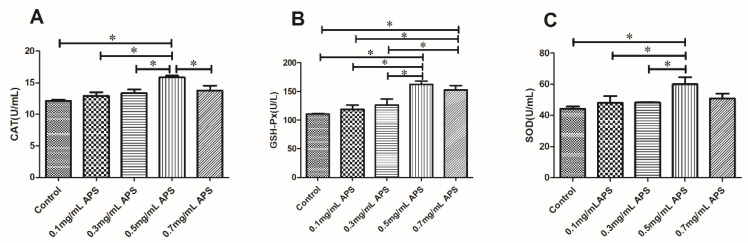
Effect of different concentrations of APS on the antioxidant enzyme activity in bull semen after thawing (**A**–**C**). The different asterisks represent significant differences among the groups (* *p* < 0.05; ** *p* < 0.01).

The results of the CAT, GSH-Px, and SOD assays after semen cryopreservation in 0, 0.1, 0.3, 0.5, and 0.7 mg/mL APS are depicted in Figure 7. The CAT, GSH-Px, and SOD levels in the APS 0.5 mg/mL group were higher (*p* < 0.05) than those in the other treatment groups.

#### 3.3.3. Effects of the Addition of Different Concentrations of AP on the Oxidation Products of Bovine Semen

**Figure 8:**
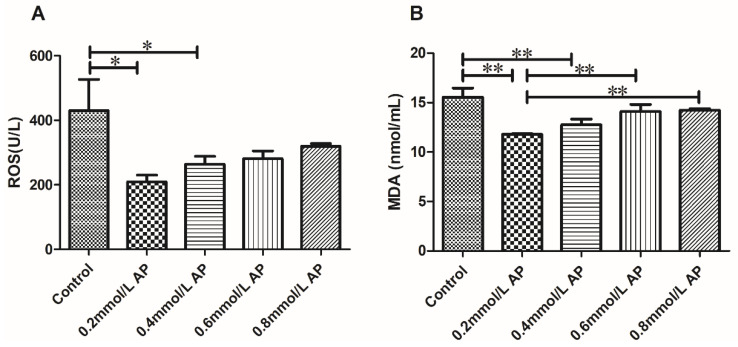
Effect of different concentrations of AP on the oxidation products of cattle semen after thawing (**A**,**B**). Different asterisk represents significant differences among the groups (* *p* < 0.05; ** *p* < 0.01).

The results of the ROS and MDA assays after sperm cryopreservation in 0, 0.2, 0.4, 0.6, and 0.8 mmol/L AP are shown in Figure 8. The ROS level was significantly reduced in the AP 0.2 mmol/L group (*p* < 0.05) compared to that in the others, and this group also demonstrated a significant reduction in MDA levels (*p* < 0.05).

#### 3.3.4. Effects of the Addition of Different Concentrations of APS on Oxidation Products of Bovine Semen

**Figure 9:**
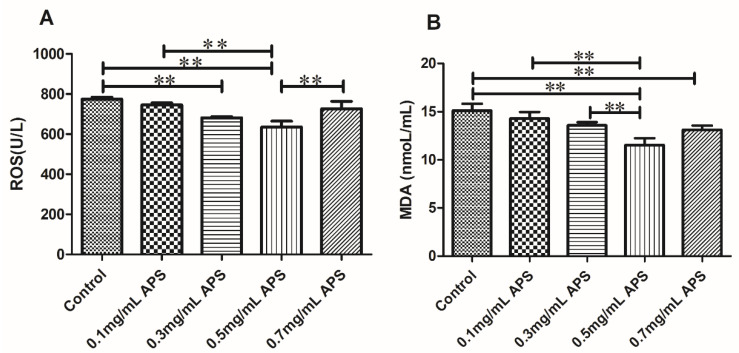
Effect of different concentrations of APS on the oxidation products of cattle semen after thawing (**A**,**B**). The different asterisks represent significant differences among the groups (** *p* < 0.01).

The results of the ROS and MDA assays in sperm samples cryopreserved in 0, 0.1, 0.3, 0.5, or 0.7 mg/mL APS are shown in Figure 9. Both the ROS and MDA levels were reduced in the APS 0.5 mg/mL groups compared to those in the others (*p* < 0.05).

### 3.4. Effect of the Combined Use of AP and APS on Several Kinematic Parameters of Bovine Semen Samples after Thawing

#### Effects of AP and APS on the Kinematic Parameters of Frozen Bovine Semen

The results are shown in Table 4. A combined addition further improved the kinematic parameters of frozen semen compared to that with 0.2 mmol/L AP alone. A combination of 0.2 mmol/L AP and 0.3 mg/mL APS significantly improved the TM, DAP, DSL, VAP, and WOB kinematic abilities. It also simultaneously relieved the VCL level (*p* < 0.05).

### 3.5. Effects of the Combined Use of AP and APS on Sperm Plasma Membrane Integrity, Acrosome Integrity, and Mitochondria Activity after Thawing

#### Effect of the Combined Use of AP and APS on Plasma Membrane Integrity, Acrosome Integrity, and Mitochondria Activity

**Figure 10:**
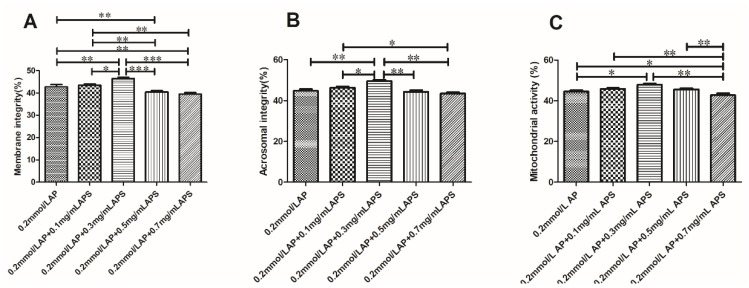
Effect of the combined use of AP and APS on the plasma membrane, acrosome, and mitochondria of bovine semen (**A**–**C**). The different asterisks represent significant differences among the groups (* *p* < 0.05; ** *p* < 0.01; *** *p* < 0.001).

A combined AP and APS treatment further improved the mitochondria activity, sperm plasma membrane integrity, and acrosome integrity in frozen semen compared to an addition of 0.2 mmol/L AP alone (Figure 10). A combination of 0.2 mmol/L AP and 0.3 mg/mL APS significantly improved the mitochondria activity, sperm plasma membrane integrity, and acrosome integrity of the semen (*p* < 0.05).

### 3.6. Antioxidant Enzymes, ROS, and MDA Content in Semen after Thawing

#### 3.6.1. Effects of the Combined Use of AP and APS on Antioxidant Enzymes in Sperm

**Figure 11:**
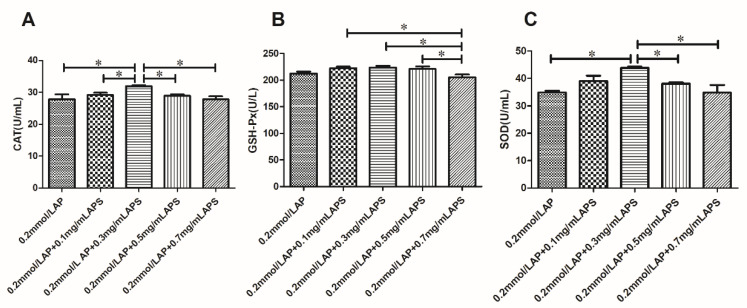
Effect of 0.2 mmol/L AP combined with each concentration of APS on the antioxidant enzyme activity after thawing of bovine semen (**A**–**C**). The different asterisks represent significant differences among the groups (* *p* < 0.05).

Combined supplementation resulted in a further improvement in the CAT, GSH-Px, and SOD activity in frozen semen when compared to 0.2 mmol/L AP supplementation alone (Figure 11). A combination of 0.2 mmol/L AP and 0.3 mg/mL APS significantly improved the SOD and CAT activity in these samples (*p* < 0.05).

#### 3.6.2. Effect of the Combined Use of AP and APS on ROS and MDA Levels

**Figure 12:**
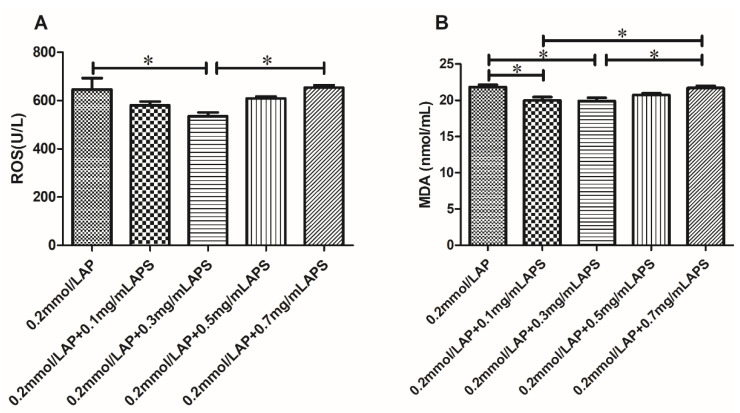
Effect of AP and APS on the oxidation products of cattle semen when used in combination (**A**,**B**). The different asterisks represent significant differences among the groups (* *p* < 0.05).

The combination of AP and APS further reduced both the ROS and MDA levels in frozen semen samples compared to 0.2 mmol/L AP alone (Figure 12), and a combination of 0.2 mmol/L AP and 0.3 mg/mL APS significantly reduced the ROS and MDA levels in these semen samples (*p* < 0.05).

## 4. Discussion

As oxidative stress affects the quality of frozen semen, adding antioxidants has become the main method used to improve sperm quality after thawing. Our results show that adding AP and APS could improve the quality of frozen semen. AP and APS were added in combination, and the quality of semen was further improved, compared to when they were added separately.

Bacterial contamination is one of the main factors causing the quality of frozen semen to decline. Recent research has shown that AP has antibacterial, antiviral, antifungal, and antiparasitic properties [25]. Consistent with the results of this study, we found that AP could reduce bacterial contamination and improve the kinematic parameters and abnormal semen movement in frozen semen. MDA is the final product of LPO. Low levels of MDA can improve the quality of frozen semen. A recent study found that AP could increase the CAT content in pig semen and reduce MDA levels [2]. In this study, it was found that adding AP could improve the CAT in frozen bovine semen and contribute to reducing the content of MDA. Overproduction of ROS can cause oxidative stress. Research shows that apigenin can show the chemopreventive potential of cancer cells by regulating the intracellular accumulation of ROS in lung cancer cells and the expression of antioxidant enzymes [26]. In this study, it was found that AP could reduce the ROS content in frozen semen and increase the expression of various antioxidant enzymes. It was also observed in previous studies that treatment with AP attenuated the reduction in superoxide dismutase activity and glutathione levels and reduced the levels of reactive oxygen species and malondialdehyde in rat hepatic stellate cells [27]. These findings are similar to the results of this study. Oxidative stress can cause sperm motility to decrease after thawing. An increasing number of studies have shown that the imbalance between the production of ROS and the level of antioxidants in sperm is the main cause of oxidative stress. As the main source of ROS is the mitochondria, mitochondrial damage caused by oxidative stress will lead to changes in the level of ROS in sperm, leading to the loss of sperm motility after thawing [28]. Mitochondria are the main energy supply sites for cells. Recent studies have found that, compared to the control group, adding APS to semen diluent can significantly increase the mitochondrial membrane potential of boar semen and reduce the level of ROS and malondialdehyde content [18]. In this study, it was found that the mitochondrial integrity of frozen semen was improved after the addition of APS, and the MDA and ROS levels were reduced. Previous research found that another way for sperm to gain energy in a diluted solution is through glycolysis. APS can provide energy for sperm in this way. It protects the mitochondria by removing reactive oxygen species and increasing the antioxidant capacity, and ultimately improves sperm energy metabolism [19]. Our results observed that the content of antioxidant enzymes such as SOD in thawed semen has been improved by the addition of APS. This result may be due to astragalus polysaccharides protecting the mitochondria of semen and improving the energy metabolism of semen. In some studies, it was found that APS could protect cells by improving cell viability, reducing apoptosis, and inhibiting the production of inflammatory cytokines [29]. APS has a protective effect on cells and can inhibit inflammatory cytokines, but its protective effect on bovine semen is unknown. It was observed that after thawing, compared to the control group, the group with APS added exhibited an increase in the vitality of sperm. Some studies have shown that APS can reduce the expression of Aspergillus toxin A, promoting apoptosis-related proteins and pro-inflammatory cytokines [30]. APS can reduce the expression of pro-inflammatory cytokines, and we found that APS can improve the inflammatory damage caused during freezing and thawing. Through observation under a microscope sperm analyzer, it was found that the multiple kinematic parameters of sperm were improved compared to the control group. The circle movement was also relieved. In some studies, APS provided effective protection in various disease models related to oxidative stress for heart, brain, kidney, intestine, liver, and lung damage [31]. A previous study showed that the use of APS in vitro can enhance sperm motility and improve testicular toxicity and has proven its potential to treat male infertility [32]. APS can not only improve the quality of semen but also has great potential in the treatment of infertility. Similar to previous studies, it was found that the addition of APS could increase the acrosome and plasma membrane integrity and other indicators of semen by adjusting the content of various antioxidant enzymes in frozen semen.

Some studies have shown that different antioxidants have a synergistic effect on the removal of active oxygen. This is because they have different binding speeds with active free radicals and different ways of action [33]. Due to the different stability that results from a combination of antioxidants, the synergistic effect of two different antioxidants can further improve the various indices of semen after thawing compared to the addition of a single antioxidant alone. As expected, our study found that combined addition of antioxidants significantly improved the quality of semen compared to a single addition, and the index with no significant difference was also improved compared to a single addition. This result confirms the feasibility of adding antioxidants in combination. In some studies, a combination of curcumin and dithioerythritol can improve the integrity of semen plasma membrane and increase the expression of glutathione peroxidase [34]. It has also been found in recent studies that, AP, as a ketone antioxidant similar to curcumin, can be used in combination with ferulic acid to improve the quality of pig semen. APS is a plant polysaccharide that is mild and non-irritating. Some studies have shown that the combined use of APS and polysaccharide peptides can immunosuppress and immunomodulate lung cancer in mice [35], resulting in low toxicity and no mutagenesis. Upon combining AP and APS, it was observed that the combination of the two antioxidants could improve the quality of semen without other side effects. DNA integrity was vital for evaluating the spermatozoa quality. Assessing the integrity of DNA is valuable for the identification of seriously damaged spermatozoa. Our experiment was mainly focus on evaluating the effects of two antioxidants on sperm motility, kinematic parameters, and various antioxidant enzyme indexes after freezing and thawing. Therefore we ignored the assessment of DNA integrity and we did not detect DNA integrity. Ignoring the detection of this intrinsic indicator is also a limitation of this experiment. In addition, adding these substances could improve the vitality after freezing; however, this does not imply that they can improve the fertilization ability, because there is no measurement of the conception rate or other indicators, which is a limitation of this research.

## 5. Conclusions

In conclusion, adding AP and APS alone could significantly improve the kinematic parameters of sperm, acrosome integrity, plasma membrane integrity, and mitochondrial activity. However, a combined addition could further improve the kinematic parameters of sperm, the integrity of the acrosome, plasma membrane, and mitochondria. These results may be due to the addition of AP and APS alleviating the level of ROS and MDA as well as increasing the enzymatic activity of CAT, SOD, and GSH-Px.

## Figures and Tables

**Table 1:** Composition of the basic hand-made extender.

Ingredient	Dosage
Glucose	1.1 g
Citric acid	1.48 g
Tris	2.42 g
Penicillin sodium	0.06 g
Streptomycin sulfate	0.1 g
Egg yolk	20%
Glycerin	3%

**Table 2:** A computer-aided sperm analysis of the kinematic parameters of frozen–thawed bull sperm after an AP addition.

	Control	0.2 mmol/L	0.4 mmol/L	0.6 mmol/L	0.8 mmol/L
TM (%)	37.30 ± 2.09 ^b^	43.25 ± 1.66 ^a^	41.43 ± 3.27 ^ab^	41.30 ± 2.09 ^ab^	38.43 ± 2.23 ^b^
DAP (μm)	7.70 ± 1.19 ^c^	11.04 ± 1.28 ^a^	10.52 ± 1.57 ^ab^	9.80 ± 1.10 ^abc^	8.38 ± 1.08 ^bc^
DSL (μm)	5.75 ± 1.04 ^b^	9.00 ± 1.23 ^a^	8.95 ± 0.72 ^a^	7.28 ± 1.39 ^ab^	7.03 ± 1.40 ^ab^
DCL (μm)	21.62 ± 1.57 ^a^	18.16 ± 1.26 ^b^	19.41 ± 0.84 ^ab^	20.63 ± 1.36 ^ab^	20.52 ± 1.60 ^ab^
VAP (μm/s)	28.23 ± 2.17 ^b^	32.69 ± 1.11 ^a^	32.61 ± 1.58 ^a^	32.00 ± 1.50 ^a^	30.59 ± 1.60 ^ab^
VSL (μm/s)	21.57 ± 1.52 ^b^	24.81 ± 1.04 ^a^	25.18 ± 1.41 ^a^	24.27 ± 0.89 ^a^	21.90 ± 1.00 ^b^
VCL (μm/s)	70.03 ± 2.78 ^a^	63.97 ± 2.28 ^b^	64.99 ± 2.98 ^b^	65.06 ± 1.45 ^b^	65.08 ± 1.29 ^b^
STR(VSL/VAP) (%)	76.99 ± 8.67	76.04 ± 4.95	77.49 ± 7.03	76.07 ± 5.42	71.77 ± 5.01
LIN (VSL/VCL) (%)	30.94 ± 3.48 ^c^	38.04 ± 1.45 ^ab^	38.86 ± 3.19 ^a^	37.32 ± 1.73 ^ab^	33.66 ± 1.62 ^bc^
WOB(VAP/VCL) (%)	40.37 ± 3.43 ^b^	51.19 ± 3.00 ^a^	50.27 ± 3.19 ^a^	49.24 ± 3.20 ^a^	47.06 ± 3.29 ^a^
ALH(μm)	3.84 ± 0.03 ^b^	4.02 ± 0.04 ^a^	3.78 ± 0.13 ^b^	3.73 ± 0.11 ^b^	3.82 ± 0.10 ^b^
BCF(hz)	10.00 ± 0.62	10.56 ± 0.77	10.11 ± 1.06	10.44 ± 1.22	9.91 ± 0.93

Abbreviations: Mean ± SEM of the total motility (TM), average path distance (DAP), straight line distance (DSL), curve distance (DCL), average path velocity (VAP), straight line velocity (VSL), curvilinear velocity (VCL), straightness (STR), linearity (LIN), wobble (WOB), amplitude of lateral head displacement (ALH), and beat/cross frequency (BCF). The different superscripted letters (a, b, c) across the rows represent significant differences (*p* < 0.05).

**Table 3:** A computer-aided sperm analysis of the kinematic parameters of frozen-thawed bull sperm after an APS addition.

	Control	0.1 mg/mL	0.3 mg/mL	0.5 mg/mL	0.7 mg/mL
TM (%)	35.50 ± 1.98 ^c^	37.24 ± 1.02 ^bc^	39.41 ± 1.87 ^ab^	40.89 ± 1.10 ^a^	38.22 ± 1.37 ^abc^
DAP (μm)	8.41 ± 0.79 ^b^	10.27 ± 1.07 ^ab^	10.78 ± 1.15 ^a^	11.45 ± 1.60 ^a^	11.10 ± 1.24 ^a^
DSL (μm)	7.27 ± 1.39 ^c^	9.02 ± 1.14 ^bc^	9.72 ± 1.00 ^ab^	11.32 ± 0.88 ^a^	10.75 ± 1.52 ^ab^
DCL (μm)	23.47 ± 0.77 ^a^	22.12 ± 1.02 ^ab^	20.52 ± 1.35 ^bc^	19.85 ± 1.29 ^c^	21.50 ± 1.28 ^abc^
VAP (μm/s)	31.83 ± 1.38 ^b^	34.45 ± 2.46 ^ab^	34.67 ± 1.28 ^ab^	36.15 ± 1.56 ^a^	34.63 ± 1.51 ^ab^
VSL (μm/s)	23.39 ± 1.44 ^b^	25.98 ± 1.45 ^a^	27.12 ± 1.16 ^a^	28.10 ± 1.55 ^a^	26.46 ± 1.18 ^a^
VCL (μm/s)	68.45 ± 1.76 ^a^	64.38 ± 1.54 ^b^	64.04 ± 1.98 ^b^	63.51 ± 2.26 ^b^	63.51 ± 2.10 ^b^
STR (VSL/VAP) (%)	73.54 ± 4.30	75.13 ± 6.17	78.94 ± 3.38	77.73 ± 2.64	76.49 ± 3.30
LIN (VSL/VCL) (%)	34.24 ± 3.00 ^b^	40.64 ± 3.02 ^a^	42.16 ± 2.31 ^a^	44.35 ± 3.62 ^a^	41.72 ± 2.45 ^a^
WOB (VAP/VCL) (%)	46.53 ± 2.38 ^b^	53.52 ± 3.76 ^a^	54.18 ± 2.33 ^a^	56.99 ± 3.07 ^a^	54.54 ± 2.06 ^a^
ALH (μm)	3.65 ± 0.17	3.55 ± 0.11	3.64 ± 0.14	3.67 ± 0.27	3.46 ± 0.19
BCF (hz)	11.11 ± 1.07	10.89 ± 0.70	11.18 ± 1.28	11.29 ± 1.29	11.23 ± 0.77

Abbreviations: Mean ± SEM of the total motility (TM), average path distance (DAP), straight line distance (DSL), curve distance (DCL), average path velocity (VAP), straight line velocity (VSL), curvilinear velocity (VCL), straightness (STR), linearity (LIN), wobble (WOB), amplitude of lateral head displacement (ALH), and beat/cross frequency (BCF). The different superscripted letters (a, b, c) across the rows represent significant differences (*p* < 0.05).

**Table 4:** A computer-aided sperm analysis of the kinematic parameters of frozen-thawed bull sperm after a combined AP and APS addition (mmol/L + mg/mL).

	0.2 AP	0.2 AP + 0.1 APS	0.2 AP + 0.3 APS	0.2 AP + 0.5 APS	0.2 AP + 0.7 APS
TM (%)	39.75 ± 1.15 ^b^	42.54 ± 1.42 ^a^	43.21 ± 1.41 ^a^	41.79 ± 1.10 ^ab^	39.52 ± 1.16 ^b^
DAP (μm)	10.31 ± 0.99 ^c^	12.55 ± 0.84 ^ab^	12.91 ± 0.98 ^a^	12.10 ± 1.16 ^ab^	10.88 ± 0.83 ^bc^
DSL (μm)	10.15 ± 1.11 ^b^	12.22 ± 0.99 ^a^	12.79 ± 1.11 ^a^	11.14 ± 0.96 ^ab^	11.00 ± 1.05 ^ab^
DCL (μm)	22.89 ± 1.67	22.15 ± 1.46	20.85 ± 1.85	22.45 ± 1.56	21.07 ± 2.18
VAP (μm/s)	33.50 ± 1.63 ^b^	35.97 ± 1.21 ^a^	36.75 ± 0.97 ^a^	34.91 ± 0.91 ^ab^	33.64 ± 1.18 ^b^
VSL (μm/s)	26.71 ± 0.78 ^b^	28.14 ± 1.15 ^ab^	29.26 ± 1.18 ^a^	27.24 ± 0.93 ^ab^	26.48 ± 2.01 ^b^
VCL (μm/s)	66.99 ± 2.83	66.59 ± 2.38	65.30 ± 2.35	66.45 ± 2.37	65.69 ± 2.60
STR(VSL/VAP) (%)	80.02 ± 5.89	78.31 ± 3.91	79.64 ± 3.69	78.12 ± 4.00	78.92 ± 7.78
LIN (VSL/VCL) (%)	39.97 ± 2.45	42.34 ± 2.74	44.82 ± 1.55	41.03 ± 1.52	40.47 ± 4.53
WOB(VAP/VCL) (%)	50.01 ± 1.37 ^c^	54.04 ± 1.45 ^ab^	56.32 ± 1.10 ^a^	52.58 ± 1.94 ^bc^	51.30 ± 2.94 ^bc^
ALH (μm)	3.46 ± 0.27	3.34 ± 0.13	3.46 ± 0.28	3.39 ± 0.20	3.26 ± 0.11
BCF(hz)	10.35 ± 1.12	10.69 ± 0.91	10.68 ± 0.99	10.62 ± 0.93	10.29 ± 1.26

Abbreviations: Mean ± SEM of the total motility (TM), average path distance (DAP), straight line distance (DSL), curve distance (DCL), average path velocity (VAP), straight line velocity (VSL), curvilinear velocity (VCL), straightness (STR), linearity (LIN), wobble (WOB), amplitude of lateral head displacement (ALH), and beat/cross frequency (BCF). The different superscripted letters (a, b, c) across the rows represent significant differences (*p* < 0.05).
